# Effects of Auditory and Visual White Noise on Oculomotor Inhibition in Children With Attention-Deficit/Hyperactivity Disorder: Protocol for a Crossover Study

**DOI:** 10.2196/56388

**Published:** 2024-08-15

**Authors:** Erica Jostrup, Marcus Nyström, Pia Tallberg, Göran Söderlund, Peik Gustafsson, Emma Claesdotter-Knutsson

**Affiliations:** 1 Child and Adolescent Psychiatry, Department of Clinical Sciences Lund University Lund Sweden; 2 Lund University Humanities Lab Lund University Lund Sweden; 3 Outpatient Department, Child and Adolescent Psychiatry Clinic Region Skåne Lund Sweden; 4 Faculty of Teacher Education Arts and Sports Western Norway University of Applied Sciences Sogndal Norway; 5 Department of Education and Special Education University of Gothenburg Gothenburg Sweden

**Keywords:** white noise, attention-deficit/hyperactivity disorder, eye tracking, cognitive performance, auditory, visual, oculomotor, child, children, protocol, crossover study, impairment, impairments, psychiatric disorders, cross modal, sensory stimulation, eye movement, noise

## Abstract

**Background:**

In attention-deficit/hyperactivity disorder (ADHD), poor inhibitory control is one of the main characteristics, with oculomotor inhibition impairments being considered a potential biomarker of the disorder. While auditory white noise has demonstrated the ability to enhance working memory in this group, visual white noise is still unexplored and so are the effects of both types of white noise stimulation on oculomotor inhibition.

**Objective:**

This crossover study aims to explore the impact of auditory and visual white noise on oculomotor inhibition in children with ADHD and typically developing (TD) children. The study will investigate the impact of different noise levels (25% and 50% visual, 78 dB auditory), and performance will be evaluated both with and without noise stimulation. We hypothesize that exposure to white noise will improve performance in children with ADHD and impair the performance for TD children.

**Methods:**

Memory-guided saccades and prolonged fixations, known for their sensitivity in detecting oculomotor disinhibition in ADHD, will be used to assess performance. Children diagnosed with ADHD, withdrawing from medication for 24 hours, and TD children without psychiatric disorders were recruited for the study.

**Results:**

Data collection was initiated in October 2023 and ended in February 2024. A total of 97 participants were enrolled, and the first results are expected between September and November 2024.

**Conclusions:**

This study will examine whether cross-modal sensory stimulation can enhance executive function, specifically eye movement control, in children with ADHD. In addition, the study will explore potential differences between auditory and visual noise effects in both groups. Our goal is to identify implications for understanding how noise can be used to improve cognitive performance.

**Trial Registration:**

ClinicalTrials.gov NCT06057441; https://clinicaltrials.gov/study/NCT06057441

**International Registered Report Identifier (IRRID):**

DERR1-10.2196/56388

## Introduction

### Background

Attention-deficit/hyperactivity disorder (ADHD) is the most prevalent neurodevelopmental disorder and one of the most common psychiatric disorders among children, affecting up to 5.3% of children around the world [1]. ADHD is characterized by developmentally inappropriate and impairing inattentiveness that may include hyperactive or impulsive behavior, highly associated with executive function deficits that cause impairments in school and difficulties with social interactions [2,3].

People with moderate to severe ADHD are commonly medicated with stimulants or nonstimulants [4], with stimulant treatment being more common due to larger effect sizes and less side effects [5]. The effects of stimulants have shown to be beneficial on several symptoms and impairments associated with ADHD, such as increased risk for other psychiatric disorders, substance use, increased risk for accidents and injuries, and antisocial behavior [6,7]. However, there are several side effects of stimulant medication; evidence for improved effects on learning processes and cognitive impairments commonly connected to ADHD varies [7,8]. Multimodal treatment, that is, medical treatment combined with nonmedical treatment and support, is thus recommended [9].

White noise exposure has been shown to improve cognitive performance in various cognitive tasks [10,11], both in neurotypical controls [12,13] and in particular for children with ADHD [14-16]. White noise is an information-dense and meaningless random signal produced by a stochastic process with a flat or constant power spectrum, that is, the same amount of energy (dB) remains over the entire frequency band [17].

To explain noise benefit among inattentive children, our point of departure is the moderate brain arousal (MBA) model proposed by Sikström and Söderlund [18]. The MBA model relies on the phenomenon of stochastic resonance (SR) where weak signals can be reinforced by external noise exposure [19]. Due to low levels of extracellular dopamine, people with ADHD have a small signal-to-noise ratio [20,21] making it hard to differentiate between the target signal and the surrounding noise [18]. By introducing the right amount of external noise in the right millisecond, through the sensory system, the noise can interact with the signal through the phenomenon of SR. It can thereby reinforce the signal and increase the signal-to-noise ratio, leading to improved cognitive performance in various tasks [14,22].

While white noise stimulation is suggested to be applicable to all modalities [18], auditory noise is the most studied and provides several demonstrations for the benefits of white noise stimulation [10,11]. For example, children with poor attention abilities or ADHD have displayed improved working memory during white noise exposure of around 80 dB [14-16]. When exposed to 70 dB white noise, an increased reading pace has also been demonstrated in this group [23]. In contrast, Allen and Pammer [24] did not find any white noise effects from noise played at “medium volume” in children with ADHD, nor did Metin et al [25] find any effects on decision-making from pink noise stimulation in the same group. When studying neurotypical adults, white noise demonstrated the ability to enhance new word learning, recognition accuracy, and word recall at 70 dB [26-28] and Awada et al [12] showed that white noise presented at 45 dB improved sustained attention, accuracy and speed of performance, while white noise presented at 65 dB improved working memory but also increased stress levels. This may indicate that different levels of auditory white noise are beneficial for different cognitive processes and may affect neurologically differentiated groups in different ways.

Other types of sensory white noise are less studied. However, in a group of children with reading disorder (RD) and phonological decoding difficulties, a common comorbidity to ADHD, visual white pixel noise stimulation has demonstrated improved reading and memory capacities [29], while a recent study using stochastic vestibular stimulation could not find any effects of white noise stimulation on visuo-spatial tasks [30].

The evidence for improved cognitive performance from cross-modal white noise stimulation is somewhat inconsistent and while working memory performance seems to be the most responsive to noise stimulation, there are other executive function deficiencies in ADHD that affect cognitive performance. Along with working memory deficits, diminished inhibitory control is a core symptom of ADHD, and also one of the most pervasive executive impairments [31]. Inhibitory control is a broad term referring to the ability to suppress nonproductive behaviors and cognitive processing. Deficiencies in inhibitory control in ADHD have been associated with several neural dysfunctions that affect different functional aspects [32-34]. For example, manual and oculomotor inhibitory controls are suggested to be independent processes, and diminished ocular inhibitory control is demonstrated to be a stronger predictor for impulsivity than manual inhibitory control [35]. Oculomotor inhibitory control has been suggested to be a more sensitive measurement in characterizing the performance of individuals with ADHD, and the use of eye tracking could thereby be a more efficient tool to study the disorder, compared with manual inhibitory control [36-38].

There are several paradigms used to assess oculomotor inhibitory control, where memory-guided saccades (MGS) and prolonged fixations (PFs) are the 2 most sensitive measurements [37,39]. In MGS, anticipatory errors reflect difficulties in inhibiting stimulus-driven responses, and even without any distractors ADHD leads to difficulties in maintaining fixation in PF [37].

### Aim and Objectives

**Figure 1:**
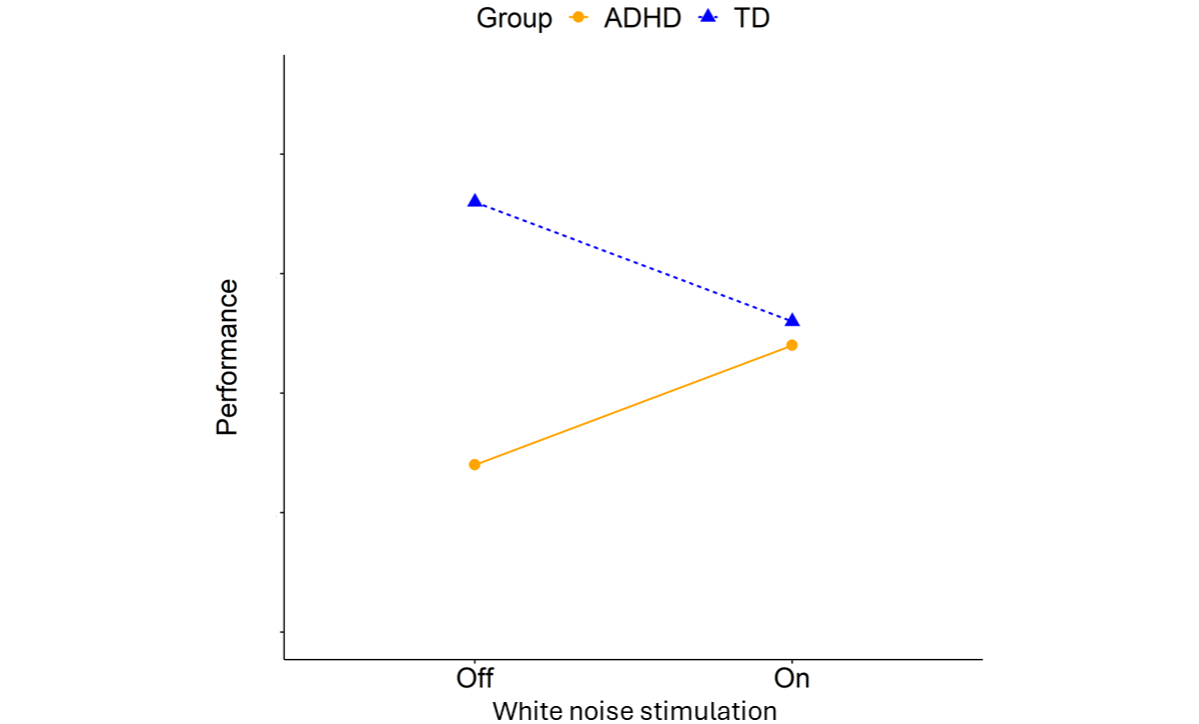
Hypotheses: (1) Children with ADHD will improve performance during noise stimulation, (2) TD children will get impaired performance by noise stimulation, (3) TD children have higher performance than ADHD children without noise stimulation, and (4) the difference between TD and ADHD will be diminished by noise stimulation. ADHD: attention-deficit/hyperactivity disorder; TD: typically developing.

With the main objective to shed further light into whether cross-modal sensory stimulation can be an effective tool to improve executive function, we here focus on eye movement control in children with ADHD. This study aims at comparing the effects of auditory and visual noise stimulation during 2 visual attention tasks. This study will be the first to investigate the effects of visual white pixel noise stimulation on children with ADHD, while the study will add to the research on auditory white noise stimulation by using a new assessment method in relation to noise stimulation. Based on the MBA model, the hypotheses are that noise stimulation will be beneficial for children with ADHD while impairing for TD children. We also hypothesize that TD children will outperform children with ADHD without noise stimulation and any such differences between TD and ADHD will be removed by the noise. The hypotheses are visualized in Figure 1.

As a secondary objective, by assessing different levels of visual white pixel noise, we expect to find an inverted U-shaped curve in accordance with the MBA model [18] and previous findings on visual noise stimulation [29].

## Methods

### Design

This crossover study has 2 groups, children with ADHD and TD children, performing MGS and PF tasks while being exposed to different levels of either auditory or visual white noise. The tasks will be performed in the order of 2 assessments of MGS (7 min), 4 assessments of PF (4 min), and ending with 2 assessments of MGS (7 min), with planned breaks in between.

During the experimental session, the participants will be exposed to 2 levels of visual noise (25% and 50%) and 1 level of auditory noise (78 dB). The noise levels are chosen based on previous research on visual and auditory noise stimulation. During auditory noise, optimal noise benefit for children with ADHD has been found in the range of 75-80 dB [14-16]. In children with RD, the optimal visual white pixel noise level has been found to be at medium levels [29]. Following an inverted U-shaped curve, too little or too much visual white pixel noise deteriorated performance for the children with RD. Here, we apply 3 levels of visual noise to enable replication of those findings.

Performance on the tasks will be evaluated during noise stimulation and compared with performance without any noise stimulation. Thus, the participants will perform each task 4 times, one in each noise condition. The order of the noise stimulation between the tasks will be randomized and counterbalanced among participants. Recruitment was active between October 2023 and February 2024.

### Study Population

The recruitment of children with an ADHD diagnosis was carried out in the outpatient child and adolescent psychiatry clinic in Lund, Sweden. Children aged 7-15 years, diagnosed with ADHD according to international guidelines following the *DSM-5* (*Diagnostic and Statistical Manual of Mental Disorders* [Fifth Edition]) [2], were asked to participate in the study. With the goal to recruit a naturalistic sample for the study, children with all types of ADHD severity and with both combined, predominantly inattentive or predominantly hyperactive-impulsive type, were included in the study. Two authors (EC-K and PT), senior consultants in child and adolescent psychiatry, confirmed all diagnoses. Participants currently on medical treatment (methylphenidate) for their ADHD had to comply to withdraw their medication for 24 hours before the study. Exclusion criteria include medicating with other substance than methylphenidate and not being willing to withdraw from medication during assessment.

TD children, without any previous record of a psychiatric disorder, in the same age range as the ADHD group, were recruited from local schools in the district. Exclusion criteria for the control group is to have a neurodevelopmental diagnosis.

Background information on all participants was collected using the SNAP (Swanson, Nolan, and Pelham) rating scale and the Five-To-Fifteen-revised (5-15R) assessment, given by legal guardians. The SNAP rating scale is an assessment tool for symptoms of inattention and impulsivity or hyperactivity in ADHD, that stems from the *DSM-IV* (*Diagnostic and Statistical Manual of Mental Disorders* [Fourth Edition]) criteria [40]. The SNAP rating scale contains 9 questions regarding inattentiveness and 9 questions regarding impulsive or hyperactive behavior that is rated on a 0 to 3 rating scale where 0=not at all, 1=just a little, 2=quite a bit, and 3=very much. The rating will be used to validate the ADHD and control groups. The 5-15R assessment consists of 181 statements concerning day-to-day situations, structured to map skills and behavioral patterns in children aged 5 to 17 years [41]. It is used for examining behavioral problems and possible developmental disorders and questions can be answered by selecting 1 of the 4 ratings from no, a little, a great deal, and very much.

### Test Battery

#### Memory-Guided Saccades

**Figure 2:**
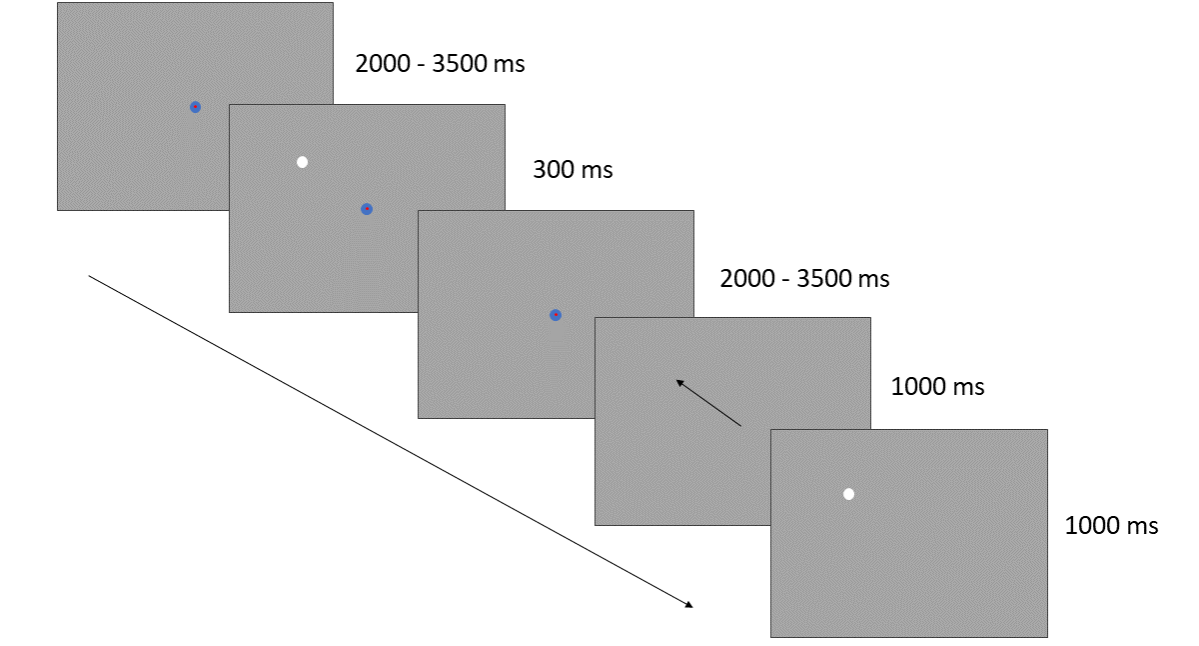
In the memory-guided saccade task, the participant will be asked to look at the central fixation point as long as it is visible and not look at a white disc but remember its placement. When the central point vanishes, the participant should move their eyes to the remembered location of the white disc as fast as possible and stay there until it reappears.

The goal of the MGS task is to focus on a central point on the screen until it disappears. The central point consists of a blue (1°) disc with a red (0.2°) disc in the middle. Following Caldani et al [42] and Mahone et al [43], the center point is visible for a randomized time interval. After 2000-3500 milliseconds, with unpredictable timing, a white disc (1°) flashes in 1 of the 4 directions on the screen for 300 milliseconds with a 10° radius from the central point. That is, the disc flash is either directed at 45°, 135°, 225°, or 315°, with the current target randomly selected in 1 of the 4 directions of the screen [44]. The participant is instructed to keep focusing on the central point until the central point disappears, 2000-3500 milliseconds after stimulus (white disc) presentation. The participant is then instructed to make a saccade to the remembered position of the white disc. After 1000 milliseconds of center point extinction, the white disc reappears for 1000 milliseconds and the participant is instructed to make a corrective saccade to the correct position (Figure 2). The assessment consists of 30 trials.

#### Prolonged Fixation

**Figure 3:**
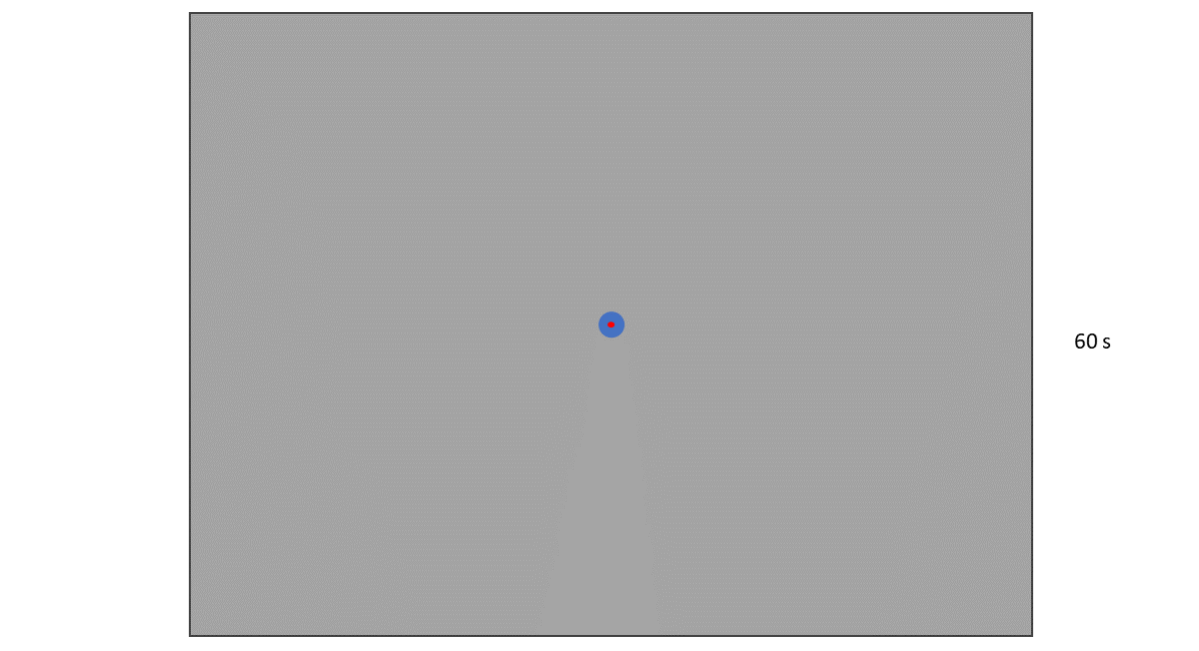
In the prolonged fixation task, the participant will be asked to focus on a central fixation point for 1 minute.

During a PF task, the participant is asked to maintain their focus on a central fixation point presented on the screen for 60,000 milliseconds (Figure 3). The central point has the same appearance as in the MGS task, that is, a blue (1°) disc with a red (0.2°) disc in the middle. The participant is instructed to return their gaze to the fixation point as soon as possible if they deviate from it.

### Intervention

#### Auditory White Noise

Auditory noise will be presented through a pair of RØDE NTH-100M earphones at 78 dB. The noise is generated from a uniform distribution U[0, 255]. To generate a stereo signal, 2 arrays drawn from such distributions are used, 1 for each channel. The audio file is sampled at 48,000 Hz, digitized to 16 bits, and saved in the .wav format. The noise level will be calibrated before each experimental session with a UNI-T UT351/352 sound level meter. Each pair of headphones will be separately calibrated when connected to the computer where the assessments are performed.

#### Visual White Pixel Noise

**Figure 4:**
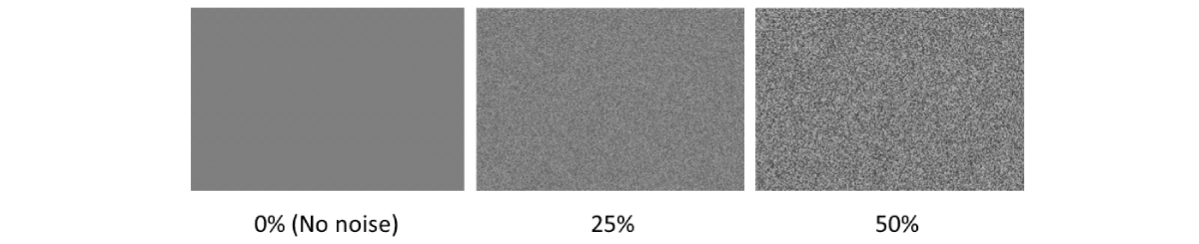
The visual white pixel noise at 0%, 25%, and 50%.

Visual noise is added to each pixel in the stimulus image by blending it with a transparent image of the same size with noise drawn from a uniform distribution U[0, 255], where 0 represents black and 255 represents white. The noise is controlled by the level of transparency of the noise image. For instance, a noise level of 100% would result in only noise and hide the stimulus image, whereas a noise level of 0% would not add any noise to the stimulus image at all. In this study, we will use noise levels of 25% and 50% (Figure 4).

### Measures and Data Collection

**Figure 5:**
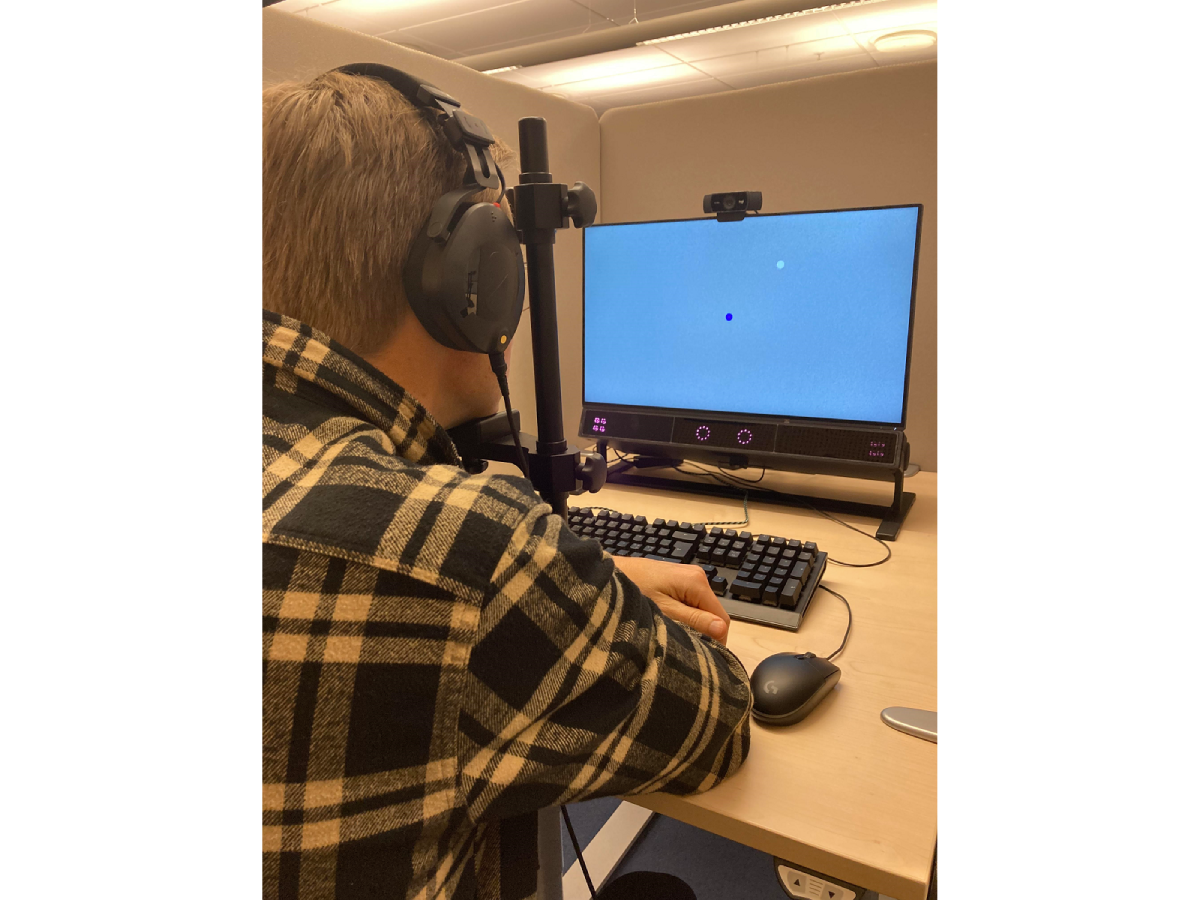
Each participant will be placed 63 cm in front of a screen and Tobii Pro Spectrum eye tracker, wear a pair of earphones, and rest their head on a chin rest during the assessments.

The study will take place at the Humanities Lab at Lund University, which has a digital classroom without any windows, equipped with 16 Tobii Pro Spectrum eye trackers (firmware: 2.6.1-orbicularis-0). Using a chin rest, participants will be placed 63 cm in front of an Eizo Flexscan EV2451 screen and be using a pair of RØDE NTH-100M earphones, connected to a desktop computer running Windows 10 (Microsoft Corp; Figure 5). Eye positions will be recorded at 600 Hz in horizontal and vertical gaze positions for both eyes, where (0,0) corresponds to the top-left on the screen and (1,1) corresponds to the low-right. The eye trackers will be calibrated with the default option in the Titta Toolbox (v.2.0.2) [45].

The dependent variables in the MGS task include (1) anticipatory saccades and (2) visuospatial working memory. Anticipatory saccades are saccades made before the extinction of the central fixation point and reflect the ability of response inhibition. Anticipatory saccades are classified as existing or nonexisting by the saccadic latency from central fixation point disappearance. Visuospatial working memory is assessed by saccadic accuracy or gain, that is, the distance between the saccade endpoint and the target. In the PF task, the dependent variable is intrusive saccades, that is, the number of deviations from the central point.

To ensure that participants understand the MGS task, a thorough presentation will be made before participants are asked to take a seat at one of the screened-off stations in the eye tracking lab. We will use similar instructions to describe the task as Mahone et al [43]: “As long as the center fixation point is visible, look only at the center point. Do not look at the flash when it occurs. When the center fixation point goes out, then immediately look to the place where you saw the flash.” All participants will then take part in a training session before data collection starts. The training measures performance by assessing correct and incorrect responses on the MGS and will be classified as successful if the participant has 3 or more correct responses, out of 5. A correct response is defined as maintaining the gaze within an area of interest of the central point until the point disappears and then moving the gaze to an area of interest of the place where the white disc was shown. If the participant has less than 3 correct responses, the test leader will give an additional instruction on how the task should be performed and the participant must redo the test. The training proceeds until the test leader is ensured that the participant understands the task.

Then, following the study design, the MGS assessment will be randomly performed in 2 of the 4 noise conditions (no noise, auditory white noise, visual white pixel noise 25%, and visual white pixel noise 50%) before a planned break. During the break, participants are instructed on the second assessment, PF, which will be performed once in each noise condition. Participants will then have a second planned break before completing the final 2 MGS assessments, securing assessments in all noise conditions. The noise conditions will be counterbalanced among participants.

To gather individual objective assessments of the tasks and interventions, participants will be asked to fill out a form inquiring about their experiences of performing the tasks. The answers will be used to examine whether there are any associations between the experiences of the different noise stimulation types and performance. The questions will focus on 3 areas and be divided into 5 questions, rated on a Likert scale. The first 2 questions will regard whether the participants experience the task as easier or harder when listening to or watching the noise. The third and fourth questions regard whether they experience the noise as uncomfortable or pleasant when listening to or watching it. The last question inquires whether they experience the tasks as boring or fun.

### Ethical Considerations

Ethical permission for the study is granted from the Swedish Ethical Review Authority (EPN 2023-02476-01) and the study is registered at ClinicalTrials.gov (NCT06057441). All legal guardians of the participating children will sign a written informed consent before the study, all children aged 14 years or less will have to orally agree to participate while children aged 15 years will leave a written informed consent. All participants were informed that discontinuing participation could be done without giving any specific reason or any following consequences. Data will be anonymized, kept confidential, and managed in accordance with the Data Protection Act, General Data Protection Regulation policies, and the Swedish Act concerning the Ethical Review of Research Involving Humans (SFS 2003:460). Data will be stored and secured both physically and electronically and will be accessible to the study team only.

### Statistical Considerations

#### Sample Size

Power calculations are based on previous studies of auditive noise effects from our group [15,29] in combination with a review on oculomotor inhibition during MGS and PF [37]. To demonstrate an improvement of stimulation with sensory noise, equivalent to half an SD on intrusive saccades on PF, approximately 15 individuals per group are required to achieve a power of 0.80 with a *P* value of .05. The study aimed at including at least 60 participants, 30 in the ADHD group and 30 TD children.

#### Data Analysis Plan

Background information will be used to classify groups in the data. SNAP scores will be used to statistically validate the TD and ADHD group while the 5-15R assessment and task evaluation will be used in an investigative order to identify other subgroups than the patient and control group. For example, reading ability and motor performance are of interest. Söderlund et al [29] identified noise benefits in children with reading disability and vestibular dysfunction has been identified as more common in children with neuropsychiatric disorders than healthy controls [46] which may have an effect on cognitive functioning [47].

The dependent variables, such as (1) anticipatory saccades in MGS, (2) visuospatial working memory in MGS, and (3) intrusive saccades in PF, will be analyzed using regression analysis and ANOVA in relation to the noise stimulation. Anticipatory saccades in MGS will be assessed through latency and classified as either correct or incorrect. All MGS onsets ≤80 milliseconds [42] from central fixation point extinction will be classified as incorrect. Saccadic accuracy will initially be classified as being correct or incorrect by the saccade being made in the right direction (1 of the 4) and then, by the gain of the saccade, that is, the amplitude of the saccade divided by the Euclidean distance from origin to target. In the PF task, saccades with an amplitude larger than 4° [48,49] will be counted as intrusive and deviant from the central fixation point. Only the left gaze position from the recorded eye tracking data will be used for the analysis. Data analysis will be conducted in R (R Core Team).

## Results

Data collection was initiated in October 2023 and ended in February 2024. A total of 97 participants were enrolled in the study. The first results are expected between September and November 2024.

## Discussion

With hypotheses grounded in the MBA model [18], this study will investigate the effects of auditory and visual white noise on oculomotor control in children with ADHD and TD children. The hypotheses are as follows: (1) noise stimulation will be beneficial for children with ADHD, (2) TD children will be impaired by noise, (3) TD children will outperform the ADHD group without any noise stimulation, and (4) noise stimulation will remove such differences.

In accordance with previous research [37,39], we expect the ADHD group to perform more anticipatory saccades, have poorer saccadic accuracy in the MGS task, and make more deviations from the fixation point in the PF task in the no-noise condition, compared with the TD children group. We also expect both noise modalities to remove the differences in performance between the groups. However, we do not have any hypotheses regarding differences between the modalities.

There has only been one previous study, to our knowledge, investigating the effects of visual white pixel noise stimulation. In a study by Söderlund et al [29], where they studied reading performance during stimulation with both auditory white noise and visual white pixel noise in children with RD, the visual white pixel noise had a larger impact on performance than the auditory noise. Since RD is a common comorbidity to ADHD, similar effects may also be found in this study. However, whether our results will be in line with these findings or not might depend on several factors.

The noise in the study by Söderlund et al [29] was applied in the same modality as the stimuli (visual), which could be more beneficial on performance than if noise and task were presented in different modalities. Here we will use only visual tasks, which, in accordance with the study by Söderlund et al [29], could potentially be affected more by visual noise than auditory noise. On the other hand, in accordance with assumptions of the MBA model, numerous experiments have shown that the SR effect works cross-modal [12,15,50]. In that sense, modality should not affect performance, only the intensity of the noise. Since noise benefit can be plotted as a reversed U-shaped curve, where too little or too much noise is detrimental to performance, the MBA model postulates that the level of noise should affect performance to a larger extent than modality [18]. Accordingly, by using several visual noise levels, that is, 0%, 25%, and 50%, we should be able to map out the inverted SR U-curve for both ADHD and TD children, as previously demonstrated by Söderlund et al [29].

Another aspect to consider when studying white noise stimulation is the nature of the task performed. Previous research on auditory white noise has found support of noise benefits on several executive functions; however, not all tasks seem to be affected [10]. Most studies have investigated working memory performance in relation to white noise stimulation and there are only 2 previous studies of white noise stimulation in relation to inhibitory control or impulsive behavior, with somewhat contradictory results. In 1 study of impulsive behavior, where children with ADHD had to choose between a smaller reward sooner or a larger reward later, no beneficial effects from noise stimulation were found [25]. On the contrary, children with ADHD displayed improved inhibitory control, as measured by performance on a go or no-go task, during auditory white noise stimulation in another study [14]. Here, we argue that the tasks used in this study are more similar to the go or no-go task than the one measuring impulsive behavior and, thus, expect to find beneficial effects of white noise stimulation in accordance with the results from Helps et al [14]. We even argue that, by assessing MGS and PF through eye tracking, we will be able to investigate the association between brain mechanisms and behavior during white noise stimulation with a high temporal resolution [51]. The method will enable measuring responses to task demands on a moment-by-moment basis and provide a more precise measure of the effects of white noise stimulation than previously used cognitive tasks [51].

The population studied also needs to be considered during noise stimulation. ADHD consists of large heterogeneity and several comorbidities [1] that could potentially affect the effects of white noise stimulation. Due to the heterogeneity, there is often large variability in psychometric test results within ADHD [52] and it is not certain that the TD group will outperform the ADHD group in the no-noise condition.

To what extent such factors affect the way external noise interacts with internal noise levels is still too early to say, since the mechanisms behind noise benefit are still unknown. However, the MBA model postulates that external white noise and internal neural noise are additive through the phenomenon of SR and that there are individual levels of optimal noise for each person. This implies that children with ADHD generally benefit from larger amounts of external noise [18]. However, not all children seem to be affected by noise stimulation. For future work, it would be valuable to investigate different subgroups of ADHD to potentially find mechanisms that would further guide the application of white noise stimulation.

This study aims at supporting the continuation of white noise research. There is often little detail on how the noise is generated in different studies and such details might affect the results. We will improve this by making the code to generate and present the noise publicly available after the study is completed.

### Conclusions

This research will offer new insights into the effects of auditory and visual white noise on oculomotor behavior in children with ADHD and TD children. The results will shed light on the potentially differential effects of auditory and visual noise on oculomotor control and working memory in both groups, with potential implications for understanding how noise can be used to improve cognitive performance.

## Abbreviations

5-15R: Five-To-Fifteen-revised
ADHD: attention-deficit/hyperactivity disorder
DSM-IV: Diagnostic and Statistical Manual of Mental Disorders (Fourth Edition)
*DSM-5*: *Diagnostic and Statistical Manual of Mental Disorders, (Fifth Edition)*
MBA model: moderate brain arousal model
MGS: memory-guided saccades
PF: prolonged fixation
RD: reading disorder
SNAP rating scale: Swanson, Nolan, and Pelham rating scale
SR: stochastic resonance
TD: typically developing

## Data Availability

The data sets generated during this study are available from the corresponding author on reasonable request.
